# Bridging Animal and Human Models: Translating From (and to) Animal Genetics

**DOI:** 10.35946/arcr.v34.3.07

**Published:** 2012

**Authors:** Amanda M. Barkley-Levenson, John C. Crabbe

**Affiliations:** **Amanda M. Barkley-Levenson***is a graduate student at the Portland Alcohol Research Center, Department of Behavioral Neuroscience, Oregon Health and Science University, and VA Medical Center, Portland, Oregon.*; **John C. Crabbe, Ph.D.,***is a professor, at the Portland Alcohol Research Center, Department of Behavioral Neuroscience, Oregon Health and Science University, and VA Medical Center, Portland, Oregon.*

**Keywords:** Alcoholism, alcohol dependence, alcohol use disorders (AUDs), alcohol research, genetic basis of alcoholism, genetics, genetic factors, phenotypes, human studies, animal models, consilience, alcohol withdrawal, alcohol sensitivity, impulsivity, dysregulated alcohol consumption

## Abstract

Genetics play an important role in the development and course of alcohol abuse, and understanding genetic contributions to this disorder may lead to improved preventative and therapeutic strategies in the future. Studies both in humans and in animal models are necessary to fully understand the neurobiology of alcoholism from the molecular to the cognitive level. By dissecting the complex facets of alcoholism into discrete, well-defined phenotypes that are measurable in both human populations and animal models of the disease, researchers will be better able to translate findings across species and integrate the knowledge obtained from various disciplines. Some of the key areas of alcoholism research where consilience between human and animal studies is possible are alcohol withdrawal severity, sensitivity to rewards, impulsivity, and dysregulated alcohol consumption.

Alcoholism is a complex disorder arising from a combination of genetic and environmental factors. The *Diagnostic and Statistical Manual of Mental Disorders, Fourth Edition* (DSM–IV) (American Psychiatric Association 1994) requires that three of seven criteria be present during a 12-month period for a diagnosis of alcohol dependence. These criteria are tolerance, withdrawal symptoms, loss of control of drinking, desire to quit, preoccupation with drinking, curtailing of other activities because of drinking, and persistence of drinking in the face of negative consequences. The use of animal models, such as rodents, nonhuman primates, and even invertebrates, allows for a degree of genetic and environmental control that would not be possible in human studies. By using these species to recapitulate discrete aspects of alcohol use disorders (AUDs) as they appear in human populations, researchers are able to target the specific biological underpinnings of the disease.

Achieving consilience between animal models and human disease is one important goal of translational research. Several years ago, a group of researchers staged a multidisciplinary meeting with the goal of identifying specific areas of alcoholism research with good potential for translation between human and animal studies (Crabbe 2010). This effort, known as the consilience project, sought to highlight both better animal models for these areas, as well as better-defined and more specific human phenotypes to target. The group focused on genetic studies because of the obvious direct translation possible across the genomes of species. Currently, animal models clearly are able to address the diagnostic criteria of tolerance and withdrawal but are less obviously capable of capturing complex emotional constructs, such as desire and preoccupation. However, behaviors such as excessive alcohol intake undoubtedly are related to AUDs, despite the fact that they do not directly lead to a diagnosis. By using various animal species to model these other behaviors and risk factors, it is possible to begin to dissect the complexities of alcoholism. After several meetings, members of the consilience project identified seven major areas for focusing translational attention (for the complete report of the consilience group, please see *Addiction Biology*, 2010, vol. 15, issue 2, entire issue). This article focuses on five of these areas, which encompass specific behavioral domains related to alcohol abuse: withdrawal, reward sensitivity, impulsivity, dysregulated alcohol consumption, and low level of response to alcohol. This article will discuss major findings from both the human and animal literature, as well as some strategies for achieving even better consilience across species in the future.

## Genetic Animal Models

Before examining the consilience of animal models and human research, it is important to briefly mention the behavioral genetic strategies used in these types of studies. Although numerous animal species are used in alcohol research, this article will focus primarily on rodent models. However, many of the approaches described here can successfully be applied to other species as well. There are three broad types of genetic methods used in rodent studies of alcohol: testing of inbred strains, selective breeding, and the creation and testing of animals with targeted genetic manipulations.

Inbred mouse and rat strains have been developed over repeated generations through brother–sister matings so that all animals within a strain are assumed to be genetically identical. As a result, these animals provide an excellent means of examining environmental contributions to alcohol-related traits because genetic variation is held constant across subjects. On the other hand, testing animals of multiple strains under standardized environmental conditions can provide evidence for the dependence of a given behavioral phenotype on genetic factors if it is found to differ across strains. Studying differences in brain morphology and neurochemistry between strains with innate differences for alcohol-related traits allows for greater insight into biological factors promoting AUDs. For example, the C57BL/6J (B6) and DBA/2J (D2) inbred mouse strains represent opposite ends of the spectrum with regard to voluntary oral consumption of alcohol, with B6 mice readily drinking large quantities and D2 mice consuming very little (e.g., Lê et al. 1994). Many studies that might explain this difference have been conducted, comparing these strains and a large panel of recombinant inbred strains derived from them for both biological and behavioral factors. Given the presumed complexity of genetic contributions to alcoholism, it is preferable to use a large number of inbred strains in order to include more genetic variation and to provide a greater ability to detect a statistical genetic correlation between traits.

Selective breeding is another method of studying genetic contributions to alcoholism. Beginning from a genetically diverse population, animals are tested for a trait of interest and are bred on the basis of their level of response. In bidirectional selection, two divergent lines are produced by breeding high responders with high responders and low responders with low responders until animals from the two lines differ significantly for the selected measure. Selective breeding is useful both for demonstrating the heritability of a trait as well as for identifying the genetic relatedness of multiple traits that might select together (known as a correlated response to selection). That is, if two lines bred for divergence on a given trait (such as alcohol preference) also differ on another measure, it can be inferred that both the selected and the correlated response share some underlying genetic contribution. For example, the numerous pairs of alcohol preferring/nonpreferring and high-alcohol–drinking/low-alcohol–drinking selected rat lines have been shown to differ on such traits as locomotor stimulation in response to alcohol and an alcohol-conditioned taste aversion, as well as on biological factors such as endogenous neurotransmitter levels (Stewart and Li 1997).

Researchers also study specific genes of interest in animal models (particularly invertebrates and mice) by targeted manipulation of the gene. This can include knockout or knockdown studies, in which the gene is removed or its expression is minimized, respectively. In another technique, transgenic experiments use increased gene expression or the insertion of a particular polymorphism or mutated version of the gene to determine the effect on the phenotype of study. Although this article will not discuss them in depth, human gene-expression and linkage studies can provide a useful method for identifying candidate genes for transgenic and knockout studies in animals (for a recent review of some human and animal gene expression techniques, see Foroud et al. 2010). In brief, gene expression profiles can be determined from samples of a variety of tissue types, including brain and blood. Although brain tissue is advantageous in demonstrating that the gene expression is likely to be behaviorally relevant, the utility of these studies is limited because they need to be conducted postmortem. Peripheral blood samples, on the other hand, are readily obtained and can be measured repeatedly in the same individuals, although the generalization of expression determined from blood samples to the brain has to be inferred. In a recent example of a translational genetic approach, researchers produced transgenic mice that expressed an ethanol-insensitive mutant form of the α2 subunit of a receptor for the neurotransmitter γ-aminobutyric acid (GABA_A_), the gene for which has been identified as a candidate gene for alcohol dependence through human studies (e.g., Reich et al. 1998). These mice showed less sensitivity to alcohol’s aversive and motor-stimulant effects than controls (Blednov et al. 2011), providing a possible behavioral mechanism for the genetic linkage of this subunit with alcohol abuse. Studies with knockout animal models must be interpreted with care, however, because the absence of genes can have profound effects on development and may result in unanticipated compensations by other systems. Increasingly, techniques are available that allow for a greater degree of spatial and temporal control of genetic manipulations (e.g., inducible knockout systems, short-interfering RNA). As technology continues to improve, these methods may provide a way to bypass the limitations of conventional knockout strategies.

### Withdrawal

Of the alcohol-related traits discussed in this article, withdrawal is the only one that also is among the DSM–IV criteria for diagnosis of an AUD. In human alcoholics, withdrawal can include both physiological and mood symptoms, with the majority of physical symptoms occurring during acute withdrawal (48 to 72 hours) (first described by Victor and Adams 1953), and emotional and mood symptoms arising in early abstinence (3 to 6 weeks) and continuing indefinitely (for review, see Heilig et al. 2010). Physical symptoms include excessive autonomic nervous system activity, central nervous system hyperexcitability, and increased seizures and convulsions, whereas mood symptoms generally consist of increased anxiety, dysphoria, and anhedonia. Historically, many theories of addiction have stemmed from a “self-medication” hypothesis, wherein continued substance abuse occurs as an attempt to prevent or relieve the experience of these negative withdrawal symptoms (Markou et al. 1998). Although current theories tend away from offering this as the only explanation, withdrawal still is considered to be a likely contributor to continued alcohol abuse and relapse.

The highly parallel nature and time course of withdrawal symptoms across species make this a key area for assessing consilience. Withdrawal severity also seems to have a genetic component, and it has been shown that alcohol withdrawal is a significant genetic factor in explaining AUD diagnoses in twin pairs (Ystrom et al. 2011). Gene polymorphisms associated with multiple neurotransmitter systems, including the dopaminergic, serotonergic, GABAergic, and opiate systems, have been explored in relation to alcohol withdrawal (Schmidt and Sander 2000). However, findings that implicate a role for certain genetic variants in withdrawal often are not replicated across studies, and there is little that can conclusively be said about the genetics underlying this trait. Difficulty in replicating results across studies likely is a result of factors such as gene-by-environment interactions and the genetic heterogeneity of the subjects and serves to highlight the complexities inherent in conducting behavioral genetic research.

A significant portion of the evidence for a genetic contribution to withdrawal has come from research using animal models. Selective breeding has produced mouse lines showing robust differences in handling-induced convulsion severity after the induction of alcohol dependence via a 72-hour vapor chamber exposure (Kosobud and Crabbe 1986). Inbred strains also exhibit differences in withdrawal severity (Metten and Crabbe 2005), and fine-mapping genetic techniques using a specialized set of B6-D2–derived strains called recombinant inbred strains helped lead to the identification of *Mpdz*^1^, a quantitative trait gene for withdrawal seizure severity (Fehr et al. 2002). This represents a significant achievement in relating human and mouse genetics because the human ortholog of this gene (*MPDZ*) has been shown to potentially contribute to alcoholism risk (Milner and Buck 2010). The behavioral significance of the seizure phenotype, however, is less clear cut because human studies thus far have failed to show a specific association between *MPDZ* and withdrawal (Karpyak et al. 2009). Tremors and seizures are observed during acute withdrawal in humans, but these physiological symptoms dissipate in later stages of withdrawal, and it currently is unknown how they may relate to the affective and other changes that occur during continued abstinence.

In recent years, the focus of withdrawal research has shifted somewhat to the mood-related symptoms of later withdrawal, such as anhedonia, dysphoria, and anxiety. Modeling emotional states in rodents proves to be more challenging than modeling seizures or central nervous system excitability. A variety of tasks exist to assess anxiety-like behavior in rodents during withdrawal, mostly based on the idea that an anxious rat or mouse will be more avoidant of situations, such as open or brightly lit areas. However, these tasks are not all influenced by the same constellation of genes in rodents (Milner and Crabbe 2008), and interpretation of data from some tasks is confounded by variations in locomotor activity (Kliethermes 2005). Increased stress reactivity during withdrawal allows researchers to study measurable physiological outcomes across species, which may be related to the negative affective symptoms of withdrawal (for review, see Breese et al. 2011). For example, blocking corticotropin-releasing hormone receptors can attenuate withdrawal-associated anxiety in rats (Gehlert et al. 2007), suggesting that the stress system might be involved in mediating anxiety that develops during abstinence. Chronic stress prior to alcohol dependence also can potentiate the anxiety-like response seen during withdrawal (Wills et al. 2010). Research in humans also has demonstrated enhanced response to negative stimuli during withdrawal (Gilman and Hommer 2008), but again the genetics remain largely unexplored.

Finally, it is important to consider that the pattern of alcohol exposure and withdrawal may be a critical factor for influencing behavioral outcomes. Studies in both humans and rodents have shown that experiencing withdrawal repeatedly can lead to a “kindling” or potentiation of both physiological and psychological withdrawal symptoms (e.g., Becker 1998; Breese et al. 2011). In addition to potentiating withdrawal symptoms, repeated cycles of induced ethanol dependence (via vapor inhalation chambers) and subsequent withdrawal seem to enhance voluntary alcohol consumption in some strains of rats and mice (e.g., Becker and Lopez 2004; Gilpin et al. 2008). This behavior generally is known as dependence- or withdrawal-induced drinking and represents an area of interest for continued consilience efforts because the genetics of this behavior have not been well explored. Some evidence suggests that genetically predisposed high-drinking animals may show greater withdrawal-associated drinking than lower-drinking animals because B6 mice show a robust effect and male mice of the high-alcohol–preferring (HAP) line show modest enhancement of drinking relative to their low-alcohol–preferring (LAP) counterpart selected line (Lopez et al. 2011). Consequently, taking into account both previous withdrawal experience and the time course of withdrawal (i.e., acute versus later withdrawal) may be a useful tool for future attempts to relate animal findings to human data.

In summary, it is possible to model specific human withdrawal symptoms in rodents rather directly, but a better understanding of which human withdrawal symptoms reflect a genetic predisposition to AUDs will help guide further success with achieving consilience in this area.

### Alcohol and Sensitivity to Rewards

Several of the current models of alcoholism include the idea of dysregulation of reward processes as a factor in the onset and maintenance of the disorder (Stephens et al. 2010). Some models propose a specific deficiency in reward sensitivity, wherein a lowered sensitivity to alcohol’s rewarding effects is thought to drive an increase in use in order to achieve the desired hedonic levels (e.g., Bowirrat and Oscar-Berman 2005). Other theories suggest more generally a dysregulation of reward processing and a hijacking of other brain systems (e.g., stress system), especially with repeated alcohol use (Koob and Le Moal 2001). In humans, the rewarding effects of alcohol most commonly are measured with self-reports. Although this is certainly an advantage of studies using human subjects (i.e., because researchers cannot directly ask a mouse how much it likes alcohol), there always is some risk of unreliability. Consequently, using both self-reports and tasks with measurable behavioral outcomes, such as willingness to work to obtain alcohol or preference for alcohol over a placebo, provides a more objective and complete measure of reward. Such assessments can be achieved through laboratory studies of self-administration and through the incorporation of behavioral economic analyses, both of which have well-developed analogues in rodent models. Biological markers of reward also are possible. One recent electrophysiological study showed evidence for altered reward processing in high-drinking (but nonalcoholic) individuals, with high-frequency drinking participants showing a greater reward-associated brain response (i.e., event-related potential) to stimuli that predicted the unexpected absence of a reward in a passive gambling task than did low-frequency drinkers (Franken et al. 2010). When considering questions of sensitivity to reward, however, it should be kept in mind that “reward” actually may represent a multifaceted sensation. That is, someone experiencing euphoria could have the same subjective sense of pleasure as someone experiencing the alleviation of anxiety, but these two outcomes may represent different actions of alcohol at the level of the brain. A single behavior that can represent two different underlying genetic substrates sometimes is called a “phenocopy;” identifying genes associated with specific behaviors can be confusing in both animal and human studies.

Despite the inference of altered reward sensitivity in AUDs, research examining the genetic contributions to this trait is relatively underdeveloped in human subjects. The dopamine neurotransmitter system is heavily implicated in regulating reward and, consequently, has been widely studied in relation to alcohol. Many studies have looked at specific alleles of the dopamine receptors that may alter reward sensitivity and subsequently either lessen or intensify the risk of developing an AUD (for review, see Le Foll et al. 2009). One of the first studies in this area suggested that the presence of one allelic form of the D2 dopamine receptor correctly predicted alcoholic status a majority of the time (Blum et al. 1990). Subsequent results have been mixed, however, with some studies failing to find any association (e.g., Gelernter and Kranzler 1999). Animal studies have provided some evidence for the role of D2 receptors: mice lacking these receptors show lower operant responding for alcohol, decreased preference for drinking alcohol, and a diminished alcohol-conditioned place preference (Cunningham and Phillips 2003). Expression of the gene encoding D2 receptors also has been shown to correlate positively with alcohol-conditioned place preference in B6-D2–derived recombinant inbred strains of mice (Hitzemann et al. 2003). In contrast, overexpression of D2 receptors in certain brain areas has been shown to decrease alcohol consumption relative to wild-type animals (Thanos et al. 2005). This highlights the difficulty of drawing conclusions about reward from behavioral measures such as alcohol intake (see the next paragraph). Consequently, although the dopamine system and its role in reward processing seem to be related to AUDs, the contributions of specific genetic variants warrant additional study.

In general, animal behavioral tasks are better developed than human ones with regard to examining alcohol reward and its possible genetic determinants. Self-administration studies are perhaps the most widely used method in this area. Mice and rats can be trained to respond operantly at a fixed ratio for infusions of ethanol or for access to a drinkable ethanol solution. In this way, it is possible to determine the ability of alcohol to maintain responding at different concentrations/doses or under different response requirements. Operant self-administration studies also can use a progressive ratio, wherein the response requirement for subsequent access to alcohol continually increases until an animal will no longer respond. The response requirement at which responding ceases to be maintained is known as the break point, and shifts in the break point are taken as an indication of differences in the rewarding effects of alcohol. As alluded to earlier, however, unambiguous assessments of the reward value of alcohol are very difficult to make using operant self-administration or home cage-drinking paradigms. An increase or decrease in responding presumably indicates a change in perceived reward, but it cannot be determined whether that change results from an increase or decrease in reward value. For example, were a given experimental manipulation to halve the operant responding for alcohol, this could indicate either that the animal now finds the alcohol to be half as rewarding as before, or that it finds it twice as rewarding and therefore only needs to administer half as much for the same perceived effect.

One widely used method for assessing reward sensitivity in animals that avoids this particular ambiguity, conditioned place preference (CPP), is based on ideas of Pavlovian conditioning. In brief, two distinct sensory cues (e.g., floor texture) are paired with either an ethanol or vehicle injection over repeated training trials. During the test, both cues are presented and the animal is allowed to choose between the two cued locations without any drug on board. A greater amount of time spent in proximity to the previously drug-paired cue suggests a drug-seeking behavior presumably resulting from rewarding effects. Alcohol-induced CPP has been shown to differ across inbred mouse strains and also between rodent lines selected for other alcohol-related traits. Mice bred for high alcohol consumption also showed greater CPP than their low-drinking counterparts (Phillips et al. 2005), and a similar relationship was found between severity of alcohol withdrawal and CPP (Chester et al. 1998). Meta-analysis of a large number of studies suggests that sensitivity to alcohol-induced CPP seems to be modestly correlated with voluntary drinking (Green and Grahame 2008). However, this relationship is not always observed. A previous study by Grahame and colleagues (2001) failed to show line differences in the ability of alcohol to condition a place preference in the HAP and LAP selected mouse lines at lower doses, whereas LAP mice showed greater preference than HAP mice at a higher dose. Furthermore, it is hard to know exactly how CPP expression relates to measures of reward in humans. Human implicit learning tests, such as the conditioned pattern-preference task, may be analogous to CPP (Johnsrude et al. 1999). This task pairs a neutral visual stimulus (e.g., monochrome pattern) with a food reward across multiple trials, while masking the pairing with a distractor memory task so that the subject is not aware of the conditioning. The subject then is presented with the paired stimulus, along with unpaired and novel stimuli, and asked to identify his or her favorite. As with CPP, a greater preference for the paired stimulus is believed to be indicative of greater sensitivity to the reward. A recent study found that self-reports of hazardous drinking were significantly correlated with stronger food-conditioned pattern preference, suggesting that repeated alcohol use might relate to increased sensitivity to nondrug reward (Balodis et al. 2010). Human conditioning tasks of this nature have not been widely implemented in the addiction field and may prove to be a promising avenue of research for relating human and animal studies of reward.

It should be noted that both CPP and self-administration paradigms also can be used to assess reward more indirectly through reinstatement procedures that aim to model drug-seeking behaviors and relapse in human alcoholics. The drug-free test session in CPP itself can arguably be seen as a measure of drug seeking, but it also is possible to test for reinstatement of place preference following extinction trials (i.e., confinement in the previously drug-paired location without a drug pairing). Place preference then can be reinstated using various manipulations (e.g., drug prime, stress, drug cues). Reinstatement of drug-paired lever pressing in operant models after extinction of the behavior also can be produced using similar methods. One key difference between animal reinstatement models and human relapse, however, is that a relapsing animal will not actually obtain any alcohol because responding on the previously drug-paired lever during reinstatement testing does not result in the delivery of alcohol (nor does the expression of a place preference result in alcohol administration). Nevertheless, drug-seeking and relapse obviously are highly relevant to the clinical treatment of AUDs, and these procedures provide a means to assess experimental manipulations or genetic factors that may prevent relapse-like behaviors following abstinence.

Despite the wide assortment of available tasks, the rewarding effects of alcohol in animal studies still must be inferred from behavioral outcomes, whereas they can be more directly reported by humans. In animals, it is not yet possible to definitively isolate “reward” as a construct separate from various contributing factors, such as subjective experience, reinforcing value, and other more nebulous inputs (Stephens et al. 2010). Nonetheless, the use of multiple approaches with animals should allow investigators to achieve convergent results. A greater focus on achieving homologous tasks between humans and animals may allow for an increased understanding of alcohol’s actions as a reinforcer, even if reward sensitivity remains somewhat elusive.

### Impulsivity

Much like alcoholism, impulsivity itself is a heterogeneous trait comprising multiple components. This can make relating the two traits even more complicated, but given the evidence for links between impulsivity and AUDs, it is worth the attempt. Impulsivity as a personality trait frequently is assessed via a variety of questionnaires, but tasks exist as well for measuring impulsive behavior (i.e., the “state” of impulsivity). Behavioral tasks seem to roughly dissociate five aspects of impulsive behavior: the inability to inhibit behavioral responses, susceptibility to distractor interference, susceptibility to proactive interference, preference for smaller immediate rewards over larger rewards after a delay, and deficits in judging elapsed time (Dick et al. 2010). However, self-reported measures of impulsivity do not always correlate well with performance on behavioral tasks (Reynolds et al. 2006). The relationship between impulsivity and alcohol use is thought to be twofold: first, a propensity toward impulsive behaviors (impulsivity as a “trait”) might coincide with a propensity toward alcohol abuse; and second, impulsive behaviors can be increased when alcohol is ingested (impulsivity as a “state”). Assessment of impulsive behavior is aided by the relatively good face validity of the tests used in both rodents and humans because many of the behavioral assays are very similar. For example, the Go/No-Go test measures behavioral inhibition and is widely used in mice, rats, and humans. This task consists of distinct cues that signal “go” trials and “no-go” trials, and a behavioral response (e.g., button push, lever press, etc.) must be made in response to the go cues and inhibited in response to the no-go cues on a series of repeated trials. Impulsive responding is characterized by responses on no-go trials. Delay-discounting procedures, which measure aversion to delayed reward, also have both human and rodent variations. These tasks offer the choice between an immediate small reward and a larger reward after a delay. By altering either the relative sizes of the rewards or the time delay to the large reward, it is possible to determine the indifference point at which the delayed and immediate rewards are valued equally. Impulsivity is associated with steeper discounting of delayed rewards (i.e., the perceived value of a delayed reward is smaller).

Despite the strong concordance in tasks across species, the genetic contributions to impulsivity remain elusive. Questionnaire-based longitudinal studies have shown that measures of impulsivity in childhood and adolescence are predictive of the development of problems with alcohol abuse later in life (Nigg et al. 2006), reinforcing the idea of similar underlying genetic risk factors. Twin studies also provide evidence for the genetic relatedness of impulsive behavior and alcohol abuse (Kendler et al. 2003). One issue in human studies that makes it difficult to tease apart the contribution of genetics to impulsivity and alcohol abuse is that many studies are conducted in people with previous drug or alcohol abuse experience. For example, it has been shown that alcohol-dependent individuals discount delayed rewards more steeply than do nondependent comparison subjects (for review, see Bickel et al. 2007), but these results can be difficult to interpret from a genetic standpoint because of the concurrent or past experience with substance use. This issue can be countered somewhat by using nonabusing subjects with a family history of AUDs and assessing them for impulsivity-related traits. One such study (Andrews et al., in press) used functional magnetic resonance imaging and found differences in the activation of brain reward circuitry during a monetary incentive delay task in individuals who had a family history of alcoholism (but who did not have a diagnosis of AUD themselves) relative to control subjects with no family history. Ideally, however, this type of study should be conducted using drug- and alcohol-naïve individuals, which can be very difficult to achieve with adult subjects. Consequently, studies often are conducted in children and adolescents at familial risk for alcoholism. Parental diagnosis with a substance use disorder has been shown to both predict behavioral disinhibition in childhood and to relate to a predisposition toward substance abuse in young adulthood (Tarter et al. 2004). Specific to alcohol abuse, a study of children of alcoholics showed greater impulsivity measures in this group relative to children with no family history of alcoholism (Dawes et al. 1997). However, despite a mean participant age of 11 years, there still were children in this study who had past experience with alcohol, tobacco, and/or other drugs, which serves to highlight how difficult it can be to conduct studies using drug- and alcohol- naïve human subjects.

An advantage of animal studies in this area, therefore, is the ability to assess impulsivity in alcohol-naïve animals of lines selected for alcohol consumption (for example) while also eliminating the complex environmental contributions that can confound human studies. This strategy has demonstrated differences in impulsivity among a variety of rodent lines differing in their level of alcohol preference. Rats of the alcohol-preferring (P) and high–alcohol-drinking (HAD) selected lines have shown a greater degree of impulsive behavior on delay discounting and behavioral inhibition tasks than their corresponding low-drinking counterparts (Steinmetz et al. 2000; Wilhelm and Mitchell 2008). A recent study in the HAP and LAP selected mouse lines found that the HAP mice showed more impulsive responding on a delay-discounting task than did the LAP mice or the progenitor strain (Oberlin and Grahame 2009). Although few attempts have been made to selectively breed for impulsivity-related phenotypes, inbred strains have been shown to differ in their impulsive behaviors, suggesting a degree of genetic control (e.g., Gubner et al. 2010). Furthermore, some measures of impulsivity have been found to be positively correlated with ethanol consumption in inbred mouse strains (Logue et al. 1998). The biggest research advantage in this area is the existence of very similar tasks across species. As with human studies, however, it may be difficult for animal studies to distinguish those aspects of impulsivity that are predisposing for AUD phenotypes from those that develop concurrently with or as a result of the disorder. As is the case with withdrawal, a continued focus on identifying the specific, well-defined facets of impulsivity that seem most important and carefully relating these behaviors both across tasks and species will be crucial for future discoveries.

### Dysregulated Alcohol Consumption

As mentioned previously, excessive alcohol consumption is not by itself a criterion for an AUD diagnosis. However, it clearly is a related behavior and is widely considered to be a key trait for any animal system purporting to be a model of disordered drinking. The consilience project group concluded that alcohol consumption further can be broken down into the components of the decision to drink or abstain, the quantity consumed, and the presence or absence of binge drinking (i.e., whether the drinking exceeds levels associated with risk of harm) (Leeman et al. 2010). All of these components then can be assessed in humans through either surveys or experimentally. A wealth of clinical and epidemiological studies have examined various aspects of alcohol consumption, generally reported as drinks per a given period of time, and numerous approaches have been used to assess genetic contributions (e.g., twin and linkage studies). For example, maximum alcohol consumption in a day by fathers has been shown to predict substance abuse in their children (Malone et al. 2002), and linkage studies have implicated high 24-hour consumption as being strongly associated with diagnosis of an AUD (Saccone et al. 2000). In addition to self-reported measures, researchers also use experimental techniques to determine alcohol consumption. These studies are helpful in that specific populations of individuals can be tested (e.g., family history positive and family history negative for AUDs), or behavioral or pharmacological manipulations can be made to determine the effect on subsequent alcohol intake. A combination of these approaches was used to show that the drug naltrexone reduces total drinks during a self-administration paradigm in those with a family history of alcoholism but has no effect on those without familial risk (Krishnan-Sarin et al. 2007). One consideration for experimental consumption studies, however, is that they usually are conducted in a laboratory setting, which may not translate directly to “real-world” drinking.

In animal models, strong evidence exists to implicate genetics as an important factor in voluntary alcohol drinking and alcohol preference: Selected mouse and rat lines have been bred for differences in alcohol consumption, and different inbred mouse strains showed marked differences in consumption measures as well. Perhaps the most classic form of drinking study in rodents presents the animal with continuous access to both an alcohol solution and water. Total consumption is measured (usually over the course of 24 hours), as is preference for or aversion to, the alcohol in relation to water. The majority of high- and low-drinking selected rodent lines have been bred for their intake on some variation of this test (for review, see Spanagel 2000). Despite their ubiquity, a common criticism of continuous-access paradigms is that there is little evidence that animals are reaching pharmacologically significant blood alcohol concentrations (BACs), even in high-drinking genotypes (Dole and Gentry 1984). Without proof that the animals actually are drinking to intoxication, it can be difficult to try to translate results back to the human condition, where intoxication is a key element. One way of promoting high BACs is by presenting alcohol only for a limited period, frequently during the animal’s circadian dark. An example of this method is the drinking-in-the-dark procedure, which generally is regarded as a model of binge drinking because animals will consume an intoxicating dose in a relatively short time period (Rhodes et al. 2005). Intake during this test has been shown to differ across inbred mouse strains, and selected lines have been bred for high BACs following the drinking period (Crabbe et al. 2009). An important consideration when interpreting drinking results is the role played by taste in mediating intake and preference. One can envision a scenario wherein an apparent genotype-dependent difference in alcohol consumption actually represents disparate sensitivity to the taste of alcohol rather than to its pharmacological effects. For example, it has been shown that although D2 mice consume very little alcohol in drinking paradigms, they will self-administer alcohol both intravenously (Grahame and Cunningham 1997) and intragastrically (Fidler et al. 2010), suggesting that their limited oral intake may be mediated at least in part by preabsorptive properties of alcohol, such as odor and taste.

In addition to modeling high alcohol consumption, researchers also have attempted to model the compulsive element of drinking that is part of the diagnostic criteria for an AUD (for review, see Vengeliene et al. 2009). “Compulsion” is a somewhat human construct that can be difficult to apply to animals. The escalation of drinking during repeated cycles of dependence and withdrawal (see withdrawal section above) may represent a shift from regulated to dysregulated drinking. Continuing to drink alcohol solutions that have been adulterated with an aversive substance such as quinine may be another example. One possible way of approaching this question is through studies using devaluation of alcohol. If an animal is trained to respond for alcohol, and alcohol subsequently is devalued through pairing with an aversive stimulus (e.g., lithium chloride injection), then continued responding for alcohol could be interpreted as being a result of a habitual or “compulsive” mechanism driving the response. A study by Dickinson and colleagues (2002) used this approach to examine potential habitual components to alcohol self-administration in rats. Rats trained to respond operantly for both food pellets and alcohol solution had either the food or the alcohol devalued with lithium chloride injections. Although devaluation decreased responding during the conditioning sessions selectively for either food or alcohol, depending on which had been paired with the injection, responding for pellets during extinction was reduced in the food-devalued group relative to control and alcohol-devalued groups. In contrast, extinction responding for alcohol was similar in both the alcohol- and food-devalued groups, although both responded at levels below that of noncontingently injected controls. These results may suggest a more rigid (“habitual”) pattern of responding for alcohol than food. Studies of this nature are an interesting avenue of research and could prove useful for enhancing the consilience between the human diagnostic criteria for AUDs and animal models of dysregulated drinking.

One barrier to better consilience for studies of drinking is the disparity between human and animal studies in how intake is reported. In human literature, intake is most commonly recorded as drinks consumed per a certain unit of time, whereas animal intake generally is measured in grams of alcohol per kilogram of body weight. This discrepancy can make it difficult to attempt to relate intake across species. In addition, human studies using self-reported consumption often lack any physiological marker of alcohol effects such as BAC. Some studies have attempted more rigorous approaches by converting reported drinks consumed to a more specific measure such as grams, or by collecting the necessary information for estimating BAC achieved (Miller and DelBoca 1994). Another method that may prove useful for relating human and animal intake is examination of the pattern of how alcohol is consumed (i.e., drinking “microstructure”). In animal studies, lickometer chambers record individual contacts made to the sipper tube and therefore provide continuous consumption data that can be analyzed for measures, such as drinking bout size, duration, or interbout interval (e.g., Ford et al. 2005). These microstructural elements potentially are analogous to similar measures taken in human laboratory studies, such as time between sips and length of time taken to finish a drink. Bout size in particular may be relevant to excessive consumption, as a “gulping” (large bout size) phenotype in primates has been shown to predict risk for heavy drinking (Grant et al. 2008). Rodent studies reinforce the genetic basis of these differences because DBA/2J mice show larger bouts than C57BL/6J mice in an intragastric self-administration procedure (Fidler et al., in press), and most high-drinking rodent genotypes seem to show greater bout size (Samson 2000).

This area of research again must confront the differences between human self-reports and voluntary animal alcohol intake. The particular difficulty of evaluating reward value from behavior is a challenge. Better and more specific definitions of intake in both animal and human studies will be crucial to future progress in this domain.

### Low Level of Response to Alcohol

As initially suggested by Schuckit and colleagues in the 1980s, a low level of response to alcohol has been thought to be a potentially predisposing factor for subsequent alcohol abuse. Schuckit evaluated family history–positive and family history–negative individuals on biological measures of alcohol sensitivity (e.g., body sway) as well as their subjective response to alcohol (e.g., self-reported “high”) and found that the family history–positive group had overall lower responsiveness to the same dose of alcohol as the family history–negative group (Schuckit 1985). Since these early studies, many more have examined variation in alcohol sensitivity as it pertains to genetics and abuse potential. Subjective response to alcohol has been one of the most widely studied measures, and multiple questionnaires exist for assessing perception of alcohol’s effects (e.g., Martin et al. 1993). These questionnaires differ with regard to whether they assess only sedative/ anxiolytic effects or if they also include measures of feelings of “activation” in response to alcohol. Although some studies of subjective response have found the same pattern of lower sensitivity in people with a family history of AUDs, others have failed to reproduce this relationship (e.g., McCaul et al. 1990). The role of low level of response in the development of AUDs remains unclear. The contradictory findings in studies of family history–positive and family history–negative individuals may be attributable to the time course of testing in relationship to when alcohol is given. Studies tend to find a low level of response in at-risk populations at peak BACs and on the descending limb of the blood alcohol curve, whereas this same group shows a greater level of response immediately after alcohol administration and during the ascending limb of the curve (for review, see Crabbe et al. 2010; Newlin and Thomson 1990). In addition to subjective response to alcohol, other biological markers of sensitivity have been studied in groups that differ for their risk of developing AUDs. For example, research shows that cortisol response after alcohol consumption is blunted in groups at risk for alcohol abuse (Schuckit et al. 1987), whereas heightened cortisol response is seen in those at low risk (Wall et al. 1994).

Many attempts have been made to model low response in animals as well. However, this is a challenging endeavor given the fact that human studies in this area rely so heavily on self-reported variables. Mild concordance with the human literature has been achieved for the cortisol response to alcohol, with a high-drinking selected rat line showing decreased corticosterone (the rodent analogue of cortisol) response to alcohol relative to the low-drinking line (Apter and Eriksson 2006). These results were contingent upon social isolation of the animals, however, and results from other alcohol-preferring lines and inbred strains have proven inconsistent (Crabbe et al. 2010). Locomotor stimulation in response to alcohol also is used in animal studies as a marker of sensitivity. The FAST and SLOW mouse lines, for example, have been bred for high and low locomotor stimulation respectively by an intoxicating dose of alcohol (Phillips et al. 2002), and FAST mice have been shown to have greater home-cage drinking than SLOW mice (Risinger et al. 1994). Differences in locomotor stimulation also have been seen in rat lines selected for drinking, with high-drinking lines being stimulated by lower doses than the low drinkers (Rodd et al. 2004). This potential genetic relationship between sensitivity to alcohol’s stimulating effects and propensity toward high consumption seems consistent with findings from the human literature that show greater self-reported stimulation to alcohol in high-risk heavy social drinkers relative to low drinkers (King et al. 2011). Many human studies, however, do not specifically address the stimulating effects of alcohol and there are fewer good behavioral endpoints for measuring this in people. A greater focus on including measures of alcohol stimulant effects in human studies will be necessary to translate the extensive locomotor stimulation animal literature across species.

Level of response to alcohol may be a good predictor of risk, but despite many years of research, there is little strict parallelism between the phenotypes studied across species, and this is the logical target for improving consilience in future studies.

## Conclusions

Ultimately, relating animal studies to those with humans requires careful consideration of the most relevant traits and tasks to be used. As seen with alcohol withdrawal, the capabilities exist to translate fine-tuned gene mapping of a behavior in mice back to people. This only proves fruitful in a clinical sense, however, if the behavior chosen is relevant to the development or expression of alcoholism in humans. Consequently, in order to continue making strides in the animal-models literature, it will be beneficial to choose the most clinically significant traits and make sure that the tasks used truly are measuring these traits. Likewise, adjustments on the human side can be made to include a greater focus on measuring a set of consistent, well-defined phenotypes that can be readily translated to animal models. For both human and animal researchers, it often can be tempting to gravitate toward tests that look similar in performance across species. However, the more important question is whether the tests are measuring and responding to the same underlying factors in both humans and animals. Designing experiments with this in mind will help lead to even greater discoveries of the genetics underlying alcohol abuse.

## Footnotes

1 By convention, gene names in animals are written in uppercase and lowercase and italicized. Gene names in humans are written in all caps and are italicized, whereas the acronyms for the encoded proteins are all caps but not italicized.
